# TLT2 Suppresses Th1 Response by Promoting IL-6 Production in Monocyte Through JAK/STAT3 Signal Pathway in Tuberculosis

**DOI:** 10.3389/fimmu.2020.02031

**Published:** 2020-09-11

**Authors:** Jinai Li, Can Cao, Yali Xiang, Zhongsi Hong, Duanman He, Haibo Zhong, Ye Liu, Yongjian Wu, Xiaobin Zheng, Huan Yin, Jie Zhou, Hanbin Xie, Xi Huang

**Affiliations:** ^1^Center for Infection and Immunity, The Fifth Affiliated Hospital, Sun Yat-sen University, Zhuhai, China; ^2^Guangdong Provincial Engineering Research Center of Molecular Imaging, Guangdong Provincial Key Laboratory of Biomedical Imaging, Department of Interventional Medicine, The Fifth Affiliated Hospital, Sun Yat-sen University, Zhuhai, China; ^3^The Third People’s Hospital of Shantou, Shantou, China; ^4^Foshan Fourth People’s Hospital, Foshan, China

**Keywords:** TLT2, Tuberculosis, monocyte, IL-6, STAT3, Th1

## Abstract

The function of triggering receptor expressed on myeloid cell-like transcript 2 (TLT2) has not been characterized and their role in pulmonary tuberculosis (TB) remains unclear. In this study, we found that surface TLT2 was up-regulated in human monocytes of patients with active TB compared to healthy subjects. *In vitro*, TLT2 expression was induced in human monocyte cell line THP-1 cells after bacillus Calmette-Guérin (BCG) or *Mycobacterium tuberculosis* (*Mtb*) H37Rv infection. Knockdown of TLT2 by siRNA transfection suppressed IL-6 expression, whereas over-expression of TLT2 increased IL-6 production in THP-1 cells infected by H37Rv. TLT2^+^CD14^+^ monocytes produced higher level of IL-6 compared to TLT2^–^ subset in active TB patients. Western blot and immunocoprecipitation revealed that TLT2 interacted with kinase JAK1/JAK2/Tyk2 to enhance STAT3 phosphorylation. Moreover, we showed that tyrosine residues 297 and 315 of TLT2 cytoplasmic domain were involved in STAT3 activation. In monocyte/CD4^+^ T cell co-culture assay, blockage of TLT2 fusion protein facilitated IFN-γ production by CD4^+^ T cells. Plate count assay showed that monocyte-mediated bacterial killing was promoted by TLT2 fusion protein. *In vivo* treatment with TLT-2 fusion protein reduced IL-6 production by macrophage but increased IFN-γ production by CD4^+^ T cell in H37Rv and BCG infected mice. Furthermore, TLT2 fusion protein attenuated inflammation, and reduced bacterial load in lung of infected mice. Together, these findings demonstrate that TLT2 negatively regulates Th1 response against mycobacterial infection, which promotes IL-6 production through JAK/STAT3 signal pathway.

## Introduction

Tuberculosis (TB) is still a serious public health threat worldwide with high morbidity and mortality (1). *Mycobacterium tuberculosis (Mtb*) can survive and persist within host macrophages to induce cells lysis that may lead to uncontrolled bacterial growth and development of chronic TB infection. Macrophages are essential in the host immune defense against mycobacteria (2). One of the mechanisms that macrophages employ to kill mycobacteria is regulated by the IFN-γ-producing Th1 cells (3). A Th1 cellular immune response is essential for optimal control of TB. Cytokines play an important role in determining the differentiation of CD4^+^ T cells into Th1 and Th2 cells. The presence of IL-4 drive precursor Th cells to differentiate into Th2, while IL-12, and IFN-γ drive Th1 differentiation (4). Additionally, IL-6 negatively regulates Th1 differentiation (5). IL-6 is a pluripotent cytokine produced by several cell types including antigen presenting cells such as macrophages and dendritic cells; and it plays an important role in the regulation of immune response during TB (6, 7). Macrophages from active TB patients (ATB) produce higher level of IL-6 than those from healthy controls (HC) (8), and elevated circulating IL-6 level of peripheral blood mononuclear cells (PBMCs) is correlated with TB disease severity (7). IL-6 produced by macrophages infected with mycobacterium species can inhibit the differentiation of naïve CD4^+^T cells into high-IFN-γ-producing effector Th1 cells (5, 9), and suppress the response of *Mtb* infected and non-infected bystander macrophage to IFN-γ (10).

Triggering receptor expressed on myeloid cells (TREM) receptors and TREM-like transcript (TLT; or TREML) receptors of the immunoglobulin superfamily are known to play an important role in innate and adaptive immune system (11). Members of the TREM family have been shown to modulate cellular response to inflammatory, pathogenic stimuli, and mediate cellular crosstalk (12). Expression of TREM-1 on blood monocytes of TB patients was enhanced, and cell-associated TREM-2 expression was increased on monocytes and lymphocytes from TB patients (13).

As compared with other conserved receptors of the TREM family, expression and function of TLT2 in the immune system are not well understood. TLT2 is highly expressed on human monocytes and B lymphocytes (14). In human, TLT2 is up-regulated in response to bacterial products, suggesting its potential role in proinflammatory response (15). A previous study in mice showed that TLT2 is highly expressed on B cells, neutrophils and macrophages, and its expression is up-regulated in response to inflammation *in vivo* on immune cells except B cells (16). TLT2 knockdown in microglia of mice leads to decreased cytokine levels in response to LPS (17). Ligation of TLT2 enhances neutrophil migration, degranulation, and the respiratory burst in response to agonists that signal via GPCRs (18). The above mentioned studies demonstrate that TLT2 is required for regulating innate immune response. However, the role of TLT2 in regulating immune response during mycobacterial infection is not yet elucidated.

In the present study, we explored a novel mechanism by which TLT2 promotes IL-6 production in monocyte. Our results demonstrate that TLT2 interacted with JAK/STAT3 and enhanced STAT3 phosphorylation. Moreover, TLT2 suppressed IFN-γ production by CD4^+^ T cells and intracellular killing of mycobacterium by promoting IL-6 production in monocyte/macrophage.

## Materials and Methods

### Ethics Statement

The study was approved by the Ethics Committee of the Fifth Hospital of Sun Yat-sen University (No.2019-L097-1). All animal experiments were performed in accordance with the National Institutes of Health Guide for the Care and Use of Laboratory Animals, and the guidelines of Animal Care and Use of Sun Yat-sen University.

### Blood Sample Collection

For healthy participants, whole blood from registered healthy blood donors (*n* = 30), were collected from the Fifth Hospital of Sun Yat-sen University (Zhuhai, China). The ATB were recruited from Infectious Disease Department, the Fifth Hospital of Sun Yat-sen University (Zhuhai, China). A total of *n* = 30 patients was screened and selected as confirmed active pulmonary TB. Eligible participants were adults older than 18 years of age with serologically confirmed HIV-negative. All patients were TB treatment naive at the time of recruitment. The exclusion criteria included patients with other known pulmonary diseases (pneumonia, cancer, and those under chemotherapy, or TB therapy). Detailed clinical characteristics and laboratory information are shown in Table 1. All study participants in this cohort were recruited at the same time.

**TABLE 1:** Characteristic of study population.

Participants	Active TB (*n* = 30)	Healthy control (*n* = 30)	*P* value
Age (mean ± SEM)	49.07 (±3.38)	41.9 (±2.28)	0.08
Male/Female	9/21	10/20	1.00
Symptom			
Fever	15 (50%)	N/A	–
Cough	28 (93.33%)	N/A	–
weakness	11 (36.67%)	N/A	–
Nasal congestion	8 (26.67%)	N/A	–
Rhinorrhea	4 (13.33%)	N/A	–
Chest pain	6 (20%)	N/A	–
Sputum smear	30 (100%)	N/A	–
Sputum culture	30 (100%)	N/A	–
TST	30 (100%)	0	–
Chest x-ray	30 (100%)	0	–
T-SPOT.TB	30 (100%)	0	–
BCG vaccinated	30 (100%)	30 (100%)	–

*N/A, no available; TST, Tuberculin skin tests; and *n* = number of participants.*

### Reagents

The following reagents were used in this study. Middlebrook 7H10 agar and Middlebrook 7H9 broth medium were purchased from BD Difco Laboratories (Sparks, MD, United States). Antibodies (Abs) against STAT3, p-STAT3 (Y705), HA tag, and DYKDDDDK tag were obtained from Cell Signaling Technology. Antibody against β-actin was obtained from Sigma-Aldrich. An inhibitor of STAT3 (Stattic) was obtained from Selleck. Recombinant human TLT2 Fc chimera and recombinant human IgG_1_ Fc were obtained from R&D Systems. Leaf^TM^ purified anti-human IL-6 antibody, purified anti-human CD126 (IL-6Rα) antibody, and IgG_1_ were obtained from BioLegend. Recombinant human IL-6 (Catalog 200-06) were obtained from PeproTech. Recombinant mouse TLT2 (Catalog CM67) were obtained from Novoprotein.

### Transient Transfection of Plasmids and siRNA

The cDNA sequences of human TLT2, STAT3, JAK1, JAK2, and Tyk2 were amplified by reverse transcription-PCR and cloned into pSG5 vector following the manufacturer’s protocol. Control siRNA and siRNAs targeting human TLT2 were purchased from RiboBio Co., LTD (Guangzhou, China). Cells were transiently transfected with 0.8 μg plasmid or 100 nM siRNA, using Lipofectamine 2000 (Invitrogen) according to the manufacturer’s instructions.

### Western Blot Analysis

Cells were washed three times with ice-cold PBS and then lysed in lysis buffer containing 1 mM phenylmethylsulfonyl fluoride, 1% (v/v) protease inhibitor cocktail (Sigma), and 1 mM DTT. Equal amounts of cell lysates were resolved by SDS-PAGE and then transferred to polyvinylidene fluoride membranes. Membranes were blocked with 5% non-fat dry milk in PBST (PBS containing 0.05% Tween 20) for 1 h at room temperature and incubated overnight with the respective primary Abs at 4°C Then, the membranes were incubated with appropriate secondary Ab at room temperature for 1 h, and at last visualized with an ECL kit (KeyGEN, Nanjing, China) according to the manufacturer’s instruction.

### ELISA for Cytokine

The protein levels of IL-6 in the culture supernatant or *in serum* were detected by ELISA using commercial ELISA kits (R&D Systems). Experiments were performed according to the manufacturer’s guidelines and quantified by the microplate reader at 450 nm.

### Real-Time PCR Analysis

Total RNA was extracted from cultured cells with TRIzol (Invitrogen) according to the manufacturer’s instructions. cDNAs were synthesized from 1 μg total RNA using RevertAid^TM^ First Strand CDNA Synthesis Kit (Termo Fisher Scientific, Waltham, MA, United States). Quantitative real-time PCR analysis was performed on Bio-Rad CFX96 real-time detection system using SYBR Green Master Mix (Applied Biosystems, Foster City, CA, United States). Data were normalized to β-actin using the delta comparative threshold cycle (Ct) method.

### Cells Sorting and T-Cell Differentiation *in vitro*

CD14^high^ cell subsets were purified by positive selection using anti-human CD14 magnetic particles (BD). Human naïve CD4^+^T cells were sorted by negative selection using human naïve CD4^+^T cell enrichment set (BD). As for *in vitro* T cell differentiation, naïve CD4^+^T cells were activated with plate-bound 1 μg/mL anti-CD3 (BD) and 1 μg/mL anti-CD28 (BD) for 3 days co-cultured with CD14^+^ cells at a 1:1 ratio. Thereafter, activated cells were stimulated with 50 nm PMA (Sigma Aldrich) and 1 μg/mL ionomycin (Sigma Aldrich) in the presence of 1 μg/mL protein transport inhibitor (BD) for 6 h before intracellular staining.

### Flow Cytometry

The cell staining procedure used in this study was previously described by Zhan and colleagues with slight modification (19). Intracellular cytokines were stained using the intracellular fixation/permeabilization buffer set (eBioscience, CA, United States). Flow cytometric analysis was performed on a FACS Fortessa flow cytometer (BD, NJ, New York, United States). Data were analyzed using FlowJo software (version 10.0.7; Tree Star).

Antibodies were purchased from the following companies. Anti-human: CD3 (BD Biosciences, briefly called BD), CD4 (BD), CD19 (BD), CD14 (BD), TLT2 (BioLegend), IFN-γ (eBioscience), IL-4 (eBioscience), IL-17A (eBioscience), IL-6 (BD), Fixable Viability Dye (eBioscience). Anti-mouse: CD3 (BD), CD4 (BD), F4/80 (eBioscience), CD11b (BD), IFN-γ (eBioscience), IL-4 (eBioscience), IL-17A (eBioscience), IL-6 (BioLegend), and TLT2 (BioLegend).

### Immunoprecipitation

Indicated constructs were transfected into HEK293T cells using lipo2000 for 24 h and then cell lysates were incubated with Anti-Flag M2 affinity gel (Sigma-Aldrich) at 4°C overnight. After washing six times with the lysis buffer, bound proteins were eluted by boiling and analyzed by Western blot.

### Bacterial Culture

*Mycobacterium bovis* bacillus Calmette-Guérin (BCG) strain 19015 and *Mtb* strain H37Rv 25618 were purchased from the American Type Culture Collection (ATCC), and was grown in Middlebrook 7H9 broth medium, or on 7H10 agar plates supplemented with 10% OADC at 37°C with an atmosphere of 5% CO2 and 95% air. For infections, BCG or H37Rv was homogenized to generate a single cell suspension. All experiments were performed at the BSL-2 and BSL-3 laboratory at Sun Yet-Sen University, with the approval from the Biological Safety Committee Board of Sun Yet-Sen University.

### Infection of Mice

C57BL/6J (B6) female mice were purchased from the Laboratory Animal Center of Guangdong Province. Mice were exposed to 1 × 10^6^ colony-forming units (CFU) of BCG or H37Rv by intravenous injection. At 21 days after infection, the bacterial titers in the lungs and spleens of each mice were determined by plate count with 7H10 agar. Infected mouse lungs were collected, fixed at 10% buffered formalin, and embedded with paraffin. Tissue sections (5 μm) were stained with hematoxylin and eosin (H&E), and examined for histopathology under an Olympus microscope (Olympus Corporation, Tokyo, Japan). All experiments were performed at the BSL-2 and BSL-3 laboratory at Sun Yet-Sen University, with the approval from the Biological Safety Committee Board of Sun Yet-Sen University.

### Statistical Analysis

The following statistical analyses were performed using Prism 5.01 (GraphPad Software, San Diego, CA, United States): One-way ANOVA with Kruskal–Wallis test or Mann–Whitney test for non-parametric tests, also, a Student’s unpaired *t*-test was used where necessary. Data are shown as mean ± SEM unless otherwise stated. A *p*-value of <0.05 was regarded as statistically significant.

## Results

### TLT2 Expression Was Up-Regulated After Mycobacterial Infection

**FIGURE 1:**
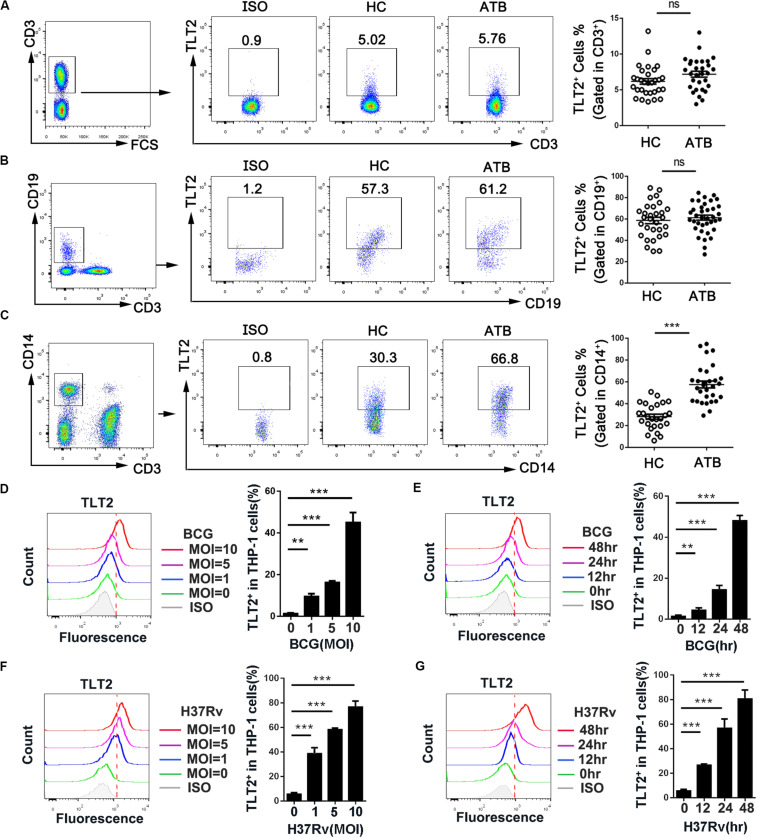
TLT2 expression was up-regulated after mycobacterial infection. PBMCs isolated from the healthy controls (*n* = 30) and active TB patients (*n* = 30) were stained with CD3-PE-Cy5.5, CD19-FITC, CD14-APC-Cy7, and TLT2-PE. T lymphocytes (CD3^+^; **A**), B lymphocytes (CD19^+^; **B**), and Monocytes **(C)** population were analyzed for TLT2 expression by flow cytometry. The percentage of TLT2^+^ cells were shown. **(D,F)** THP-1 cells were infected with BCG or H37Rv at an MOI of 0, 1, 5, 10 for 24 h. Representative flow cytometric plots and the percentage of TLT2 positive THP-1 cells were shown. **(E,G)** THP-1 cells were infected with BCG or H37Rv at an MOI of 5 for 0, 12, 24 and 48 h. Representative flow cytometric plots and the percentage of TLT2 positive cells were shown. Data are shown as the mean ± SEM of three independent experiments that yielded similar results. HC, healthy controls; ATB, active TB; ns, no significance; **p < 0.01; and ***p < 0.001.

Although TLT2 is up-regulated in response to inflammation (16), the expression and function of TLT2 in *Mtb* infection remains to be studied. To determine whether TLT2 can respond to mycobacterial infection, we examined PBMCs isolated from HC and ATB and analyzed the expression of TLT2 by flow cytometry. TLT2 expression in T cells and B cells had no significantly difference between two groups (Figures 1A,B). TLT2 was highly expressed in CD14^+^ monocytes of ATB compared with HC (Figure 1C). The frequency of TLT2^+^ CD14^+^ cells in ATB was about 57% (57.63 ± 3.14), while in HC, it was 28% (28.17 ± 2.33). To confirm the expression of TLT2 in response to mycobacterial infection *in vitro*, THP-1 cells were infected with BCG or H37Rv, and the expression of TLT2 was analyzed by flow cytometry. We found that the expression of TLT2 in BCG-, and H37Rv-infected THP-1 cells were increased in a time- and dose-dependent manner (Figures 1D–G). TLT2 expression was significantly increased *in vitro* after mycobacterial infection, suggesting that TLT2 was involved in immune response against mycobacterial infection.

### TLT2 Promoted IL-6 Expression in Monocyte After *Mtb* Infection

**FIGURE 2:**
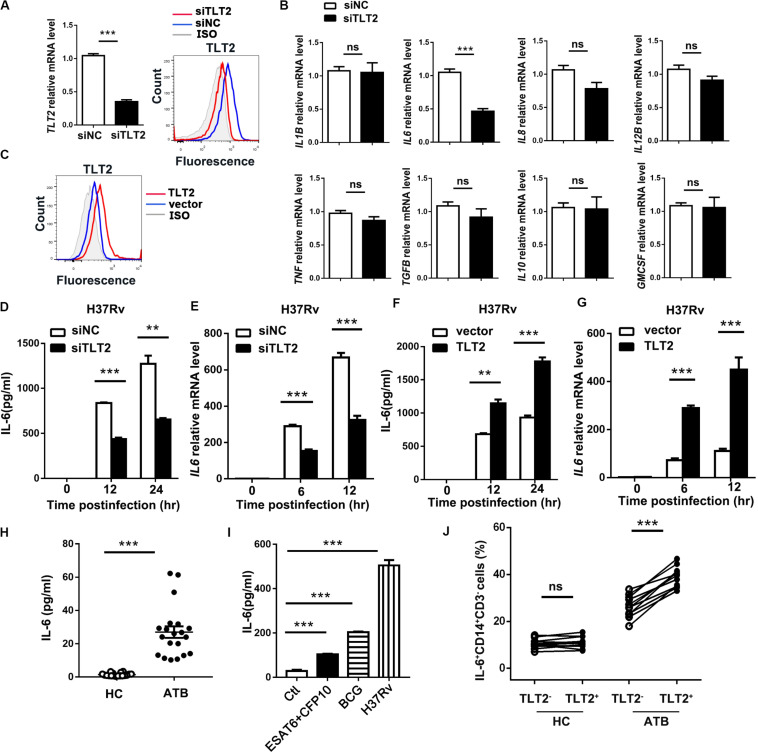
TLT2 promoted *Mtb*-induced IL-6 expression. **(A)** THP-1 cells were transfected with negative control siRNA (siNC) and TLT2 siRNA (siTLT2) for 24 h. The relative mRNA level of *TLT2* were examined by real-time PCR and the protein level of TLT2 were examined by flow cytometry. **(B)** TLT2 silenced with siRNA in CD14^+^ monocytes for 24 h, followed by H37Rv infection at an MOI of 5 for 12 h. The mRNA levels of *IL1B, IL6*, *IL8*, *IL12B*, *TNF*, *TGFB*, *IL10*, and *GMCSF* were determined by real-time PCR. **(C)** THP-1 cells were transfected with empty vector or TLT2 plasmid for 24 h. The protein levels of TLT2 were analyzed by flow cytometry. **(D,F)** THP-1 cells were infected with H37Rv at an MOI 5 for 0, 12, and 24 h after transfected with siRNA or plasmid. The protein levels of IL-6 in the culture supernatant were measured by ELISA. **(E,G)** THP-1 cells were infected with H37Rv at an MOI 5 for 0, 6, 12 h after transfected with siRNA or plasmid. The mRNA levels of *IL6* were detected by real-time PCR. **(H)** Levels of serum IL-6 in active TB patients and healthy controls were determined by ELISA. **(I)** Human PBMCs from healthy controls were treated with ESAT-6/CFP10 (5 μg/ml), BCG (MOI = 5), or H37Rv (MOI = 5) for 24 h. IL-6 levels in the culture supernatants were determined by ELISA. **(J)** Expression of IL-6 on CD3^–^CD14^+^ cells of PBMCs isolated from HC and ATB were determined by flow cytometry. Percentage of IL-6 expressing CD3^–^CD14^+^ cells in the HC (*n* = 11) and ATB (*n* = 11) were shown. Data are shown as the mean ± SEM of three independent experiments. ns, no significance; **p < 0.01; and ***p < 0.001.

To examine the mechanism of TLT2 on mycobacterial infection triggered cytokine production, specific siRNAs against TLT2 (siTLT2) were used to suppress the expression of TLT2, and the silencing efficacy was detected by real-time PCR and flow cytometry. Relative mRNA level and the protein level of TLT2 were decreased in siTLT2-treated groups (Figure 2A). Knockdown of TLT2 in monocytes followed by H37Rv infection impaired *IL6* production apparently compared with other cytokines (Figure 2B). The protein and the mRNA level of H37Rv-induced IL-6 were significantly impaired after silencing TLT2 (Figures 2D,E). Additionally, we established the expression of plasmid for human TLT2, the overexpression level of TLT2 was detected by flow cytometry (Figure 2C). IL-6 expression was significantly increased after TLT2 overexpression followed by H37Rv infection (Figures 2F,G). Several studies have reported increased IL-6 serum concentrations in TB (20–23). Consistent with that, we found that IL-6 was significantly elevated in serum from TB patients (Figure 2H). Early secreted antigenic target of 6 kDa (ESAT-6) and culture filtrate protein 10 kDa (CFP10) of *Mtb* are essential virulence factors. Human PBMCs from HC were treated with ESAT-6/CFP10, BCG, or H37Rv for 24 h. Our results showed that BCG induced higher IL-6 production than ESAT-6/CFP10, while H37Rv induced higher IL-6 production than BCG (Figure 2I). To confirm the relationship between TLT2 and IL-6, we examined PBMCs of human subjects. The percentage of IL-6 expressing monocytes was significantly increased in TLT2^+^ cells compared with TLT2^–^ cells of ATB, and the IL-6 expression level was higher in ATB than HC (Figure 2J). Overall, data suggested that the up-regulation of TLT2 on monocytes triggered IL-6 production following *Mtb* infection.

### TLT2 Promoted IL-6 Expression Through the Activation of STAT3

**FIGURE 3:**
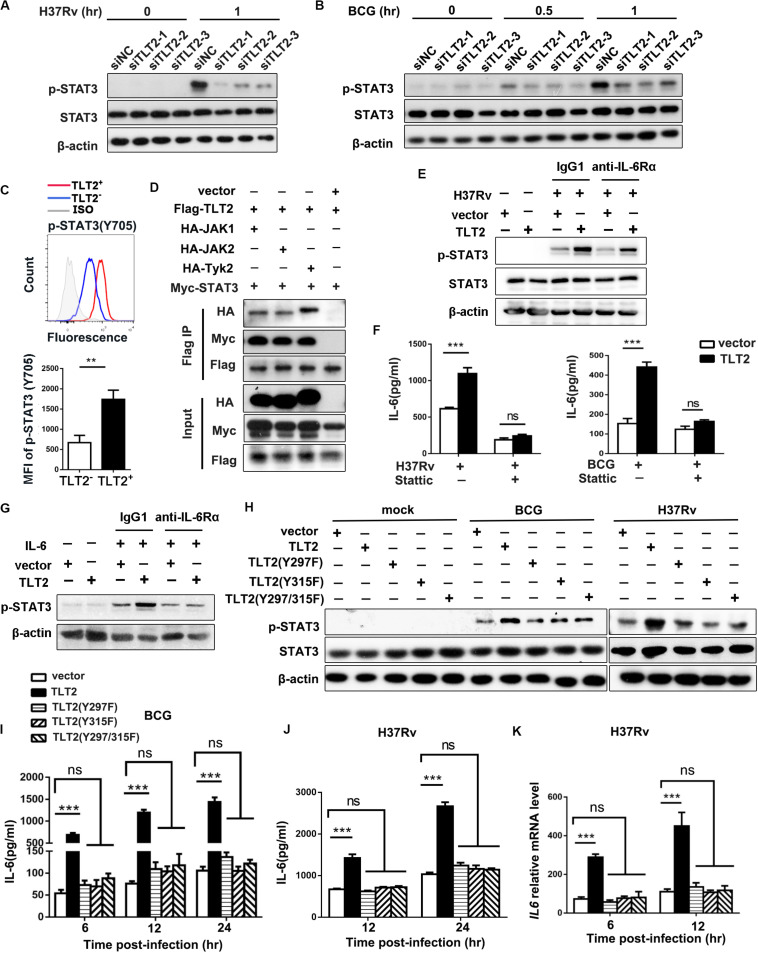
TLT2 promoted IL-6 expression via the induction of STAT3 phosphorylation. THP-1 cells were transfected with siNC and siTLT2, followed by H37Rv **(A)** and BCG infection **(B)** at an MOI of 5; and the phosphorylation of STAT3 and total STAT3 were determined by Western blot. **(C)** STAT3 (Tyr705) phosphorylation in TLT2^+^ or TLT2^–^ CD14^+^ monocytes of active TB patients were analyzed by flow cytometry and the MFI of phosphorylated STAT3 was quantified. **(D)** Co- immunoprecipitation assay of the interaction between the TLT2 and STAT3 when 293T cells were co-transfected with JAK kinase. **(E)** After overexpression of TLT2 for 24 h, THP-1 cells were incubated with either IgG1 or anti-IL-6Rα (1 μg/ml) for 1 h prior to H37Rv infection at an MOI of 5 for 1 h. Phosphorylation of STAT3 was determined by Western blot. **(F)** After transfecting with empty vector or TLT2 plasmid for 24 h, THP-1 cells were treated with STAT3 inhibitors Stattic (5 μm) for 1 h before infection with H37Rv or BCG at an MOI of 5 for 24 h, and the protein level of IL-6 in the culture supernatant was measured by ELISA. **(G)** After overexpression of TLT2 for 24 h, THP-1 cells were incubated with either IgG1 or anti-IL-6Rα (1 μg/ml) for 1 h prior to addition of IL-6 at 100 ng/ml for 1 h. Phosphorylation of STAT3 was determined by Western blot. **(H)** TLT2 and its Y297F, Y315F, or Y297/315F mutants were transiently overexpressed as indicated in THP-1 cells followed by BCG and H37Rv infections at an MOI of 5 for 1 h, and phosphorylation of STAT3 and total STAT3 were determined by Western blot. **(I)** IL-6 production in the culture supernatant was measured by ELISA after TLT2 and its Y297F, Y315F, or Y297/315F mutants were transiently overexpressed as indicated in THP-1 cells followed by BCG infection at an MOI of 5 for 6, 12, 24 h. **(J)** IL-6 production in the culture supernatant was measured by ELISA after TLT2 and its Y297F, Y315F, or Y297/315F mutants were transiently overexpressed as indicated in THP-1 cells followed by H37Rv infection at an MOI of 5 for 12, 24 h. **(K)** The *IL6* expression was determined by real-time PCR after TLT2 and its Y297F, Y315F, or Y297/315F mutants were transiently overexpressed as indicated in THP-1 cells followed by H37Rv infection at an MOI of 5 for 6 and 12 h. MFI, mean fluorescence intensity. Data are shown as the mean ± SEM of three independent experiments that yielded similar results. ns, no significance; **p < 0.01; ***p < 0.001.

To determine the mechanism by which TLT2 promote IL-6 expression, we examined the activation of Janus kinase (JAK)–signal transducer and activator of transcription (STAT) pathway. ESAT-6 of *Mtb* has been shown to stimulate macrophage IL-6 production through STAT3 activation (24). Infection-induced STAT3 phosphorylation in THP-1 cells were diminished after silencing TLT2 followed by H37Rv or BCG infection (Figures 3A,B), suggesting that STAT3 was required for TLT2 function on cytokine secretion. In PBMCs from ATB patients, higher levels of phosphorylated STAT3 (Tyr705) were detected by flow cytometry in TLT2^+^CD14^+^ monocytes compared to TLT2^–^CD14^+^ monocytes (Figure 3C). STAT3 can be phosphorylated via the receptor in a JAK kinase-dependent manner or through the intrinsic receptor tyrosine kinase domains (25). We examined the interaction between TLT2 and STAT3 by co-immunoprecipitation (IP) assay. JAK1, JAK2, and Tyk2 were co-immunoprecipitated by TLT2 in transfected 293T cells. Moreover, STAT3 interacted with TLT2 in transfected 293T cell in the presence of JAK kinase (Figure 3D). To distinguish between effects on STAT3 phosphorylation that is TLT2-derived or due to IL-6 changes, THP-1 cells overexpressing TLT2 were incubated with either IgG1 or anti-IL-6Rα for 1 h prior to H37Rv infection. Our results showed that up-regulation of STAT3 phosphorylation due to TLT2 overexpression following H37Rv infection was not blocked by pretreatment with anti-IL-6Rα (Figure 3E). Our data indicated that STAT3 activation is directly downstream in the signaling of TLT2. To verify the role of STAT3 on TLT2-regulated IL-6 production, we applied a specific STAT3 inhibitor, Stattic. Pretreatment of THP-1 cells with Stattic reduced H37Rv- or BCG-induced IL-6 secretion. The enhancement of IL-6 resulted from TLT2 overexpression was reversed after Stattic treatment (Figure 3F). In addition, TLT2 also promoted IL-6-induced STAT3 phosphorylation, which was blocked by pretreatment with anti-IL-6Rα (Figure 3G). Therefore, TLT2 didn’t serve as a receptor of IL-6. Overall, data suggested that TLT2 promoted IL-6 expression through the activation of STAT3, also specific tyrosine residues on the TLT2 were phosphorylated by activated JAKs, and served as docking sites for STAT3 resulting in subsequent IL-6 production.

### Tyrosine Residues 297 and 315 of TLT2 Were Involved in STAT3 Activation

The predicted transmembrane domain of TLT2 does not contain any charged amino acids needed for association with the adaptor molecule DAP12 (26). On the contrary, the short cytoplasmic tail region of TLT2 protein contains two tyrosine, Y297 and Y315, which has been reported to form the motif contributing to the recruitment and activation of STAT3 (27–29). We single and double mutated these two tyrosine residues into phenylalanine. The up-regulation of STAT3 phosphorylation due to the TLT2 overexpression following BCG and H37Rv infection was absent in the overexpression of TLT2 single Y297F or Y315F mutant and Y297/315F double mutant in the THP-1 cells (Figure 3H). These data indicated that both Y297 and Y315 of the TLT2 cytoplasmic domain played a role in the STAT3 activation serving as docking sites for STAT3. The mRNA and protein levels of IL-6 were up-regulated in THP-1 cells after TLT2 overexpression, but this enhancement was abrogated by the TLT2 tyrosine mutants (Figures 3I–K). Taken together, data suggested that the tyrosine residues 297 and 315 of TLT2 were required for STAT3-induced IL-6 expression.

### TLT2 Expressed on Monocytes Inhibited Th1 Response Dependent on IL-6 *in vitro*

**FIGURE 4:**
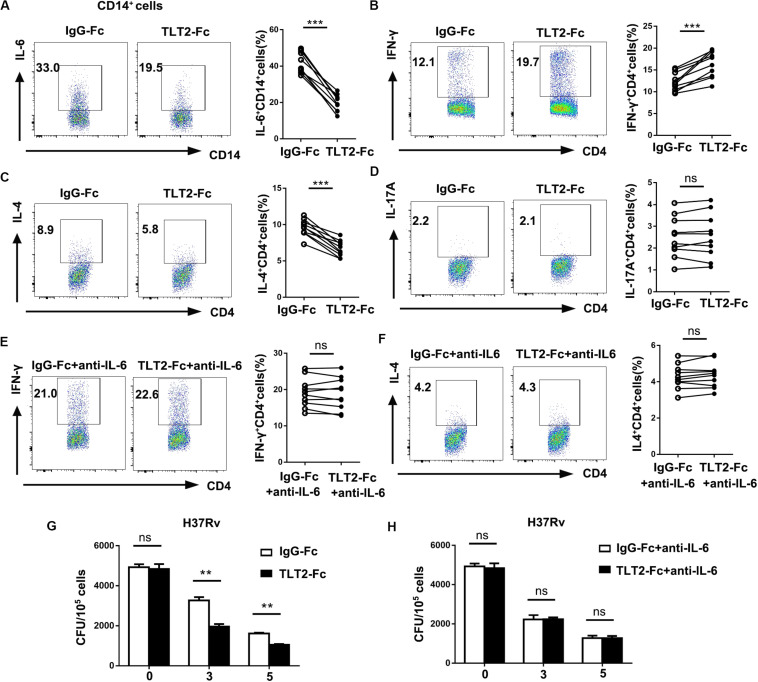
TLT2 expressing monocytes negatively regulated Th1 response against *Mtb* infection. **(A)** CD14^+^ monocytes isolated from PBMCs of the healthy controls were treated with recombinant chimeric human TLT2-Fc (4 μg/mL) and human IgG1-Fc (4 μg/ml) for 24 h followed by H37Rv infection at an MOI of 5 for 12 h. The TLT2^+^ CD3^–^ CD14^+^ cells were analyzed for the expression of IL-6 by flow cytometry. **(B–D)** Naïve CD4^+^ T cell purified from human PBMCs of healthy controls using magnetic separation. CD4^+^ T cell were activated with plate-bound anti-CD3 (1 μg/ml) and anti-CD28 (1 μg/ml) for 3 days co-culture with CD14^+^ monocytes in the presence of recombinant chimeric human TLT2-Fc (4 μg/ml) and human IgG1-Fc (4 μg/ml). CD14^+^ monocytes were pre-incubated with heat-killed H37Rv followed by co-cultured with CD4^+^ T cell. IFN-γ **(B)**, IL-4 **(C)**, and IL-17A **(D)** expressions in CD4^+^ T cells were measured by flow cytometry, representative flow cytometric plots and the percentages of cells were shown. **(E,F)** Naïve CD4^+^ T cell co-cultured with CD14^+^ monocytes as described in **(B)**, but in the presence of neutralizing anti-IL-6 mAbs (10 μg/ml; anti-IL-6). IFN-γ and IL-4 expressions in CD4^+^ T cells were measured by flow cytometry, representative flow cytometric plots and the percentages of cells were shown. **(G)** Monocytes were infected with H37Rv at an MOI of 10 for 2 h. After washing, H37Rv -infected monocytes were cultured with T cells in the presence of anti-human IgG1-Fc, or anti-human TLT2-Fc. CFU were calculated on Day 0, 3, 5. **(H)** H37Rv-infected monocytes were cultured with T cells as described in **(G)**, but in the presence of anti-IL-6 mAbs. CFU were calculated on Day 0, 3, 5. Data are a representative of at least three independent experiments. ns, no significance; **p < 0.01; and ***p < 0.001.

Previous studies implied that IL-6 produced by macrophages infected with mycobacterium species suppresses T cell response (9), and inhibits macrophage response to IFN-γ (10), while promotes Th2 T cell differentiation by activation of NFAT and induction of early IL-4 gene (30). As TLT2 promotes IL-6 expression through the activation of STAT3, we determined the role of TLT2 on T cell response. Treatment with recombinant chimeric human TLT2-Fc led to the reduction of H37Rv- or BCG-induced IL-6 derived from TLT2^+^ monocytes compared with human IgG1-Fc, which revealed that TLT2-Fc partially inhibits TLT2 signal (Figure 4A and Supplementary Figure 1A). Monocytes and naïve CD4^+^ T cells were isolated from healthy donors and co-cultured for 3 days. TLT2-Fc stimulation directed the differentiation of CD4^+^T cells to a Th1 phenotype since the cells produced high amounts of IFN-γ, but reduced Il-4 production (Figures 4B,C and Supplementary Figures 1B,C). TLT2-Fc stimulation had no significant effect upon Th17 differentiation (Figure 4D and Supplementary Figure 1D). Moreover, addition of anti-IL-6 mAbs inhibited TLT2-Fc mediated T cell differentiation (Figures 4E,F and Supplementary Figures 1E,F). TLT2-Fc stimulation mediated monocyte IL-6 reduction resulted in elevated Th1 cell differentiation but Th2 down-regulation. Blocking TLT2 expression with TLT2-Fc decreased CFU of H37Rv or BCG compared to IgG-Fc and addition of anti-IL-6 mAbs restored CFU (Figures 4G,H and Supplementary Figures 1G,H). In summary, TLT2 expressed on monocytes suppressed Th1 response through an IL-6 dependent mechanism.

### TLT2 Inhibited Th1-Mediated Host Defense Against Mycobacterial Infection *in vivo*

**FIGURE 5:**
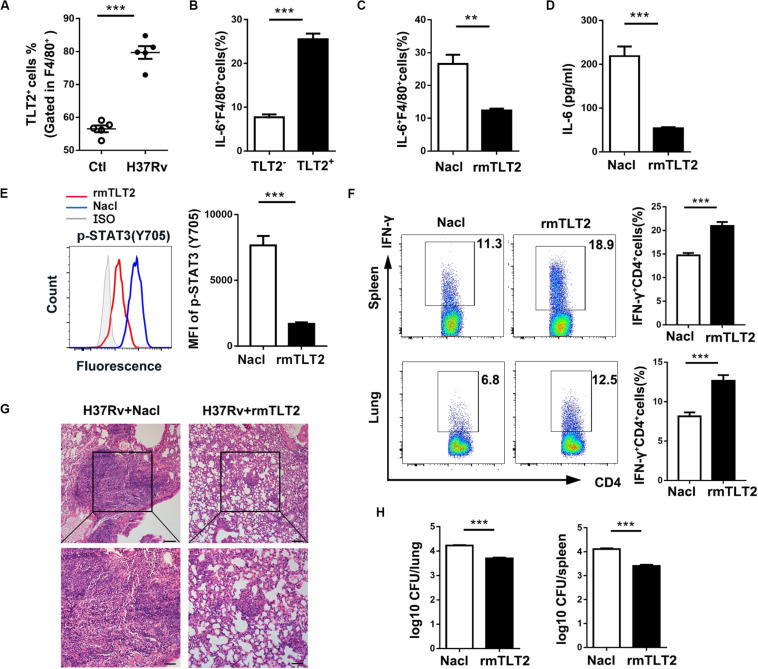
TLT2 inhibited Th1-mediated host defense against *Mtb* infection *in vivo*. **(A)** C57BL/6 mice were intravenously (i.v.) injected with 1 × 10^6^ CFU of H37Rv. The proportion of TLT2^+^F4/80^+^ cells was assessed by flow cytometry in the spleens at 21 days post-infection. **(B)** IL-6 expression of spleen in TLT2^+^ or TLT2^–^F4/80^+^ cells were analyzed by flow cytometry. Mice were intraperitoneally (i.p.) injected with recombinant mouse TLT2 (rmTLT2) for 24 h, followed by intravenous (i.v.) H37Rv infection. At 21 days post infection, the lungs and spleens were collected and analyzed for the following tests. **(C)** The frequency of splenic IL-6^+^ F4/80^+^ cells were determined by flow cytometry. **(D)** Protein level of IL-6 in serum was determined by ELISA. **(E)** STAT3 (Tyr705) phosphorylation in F4/80^+^ cells were analyzed by flow cytometry. **(F)** Percentages of IFN-γ producing splenic and pulmonary CD4^+^ T cells were analyzed by flow cytometry. **(G)** Lung sections were stained with hematoxylin-eosin (H&E) and checked for histopathology under microscope. **(H)** Bacteria burden in the lungs and spleens were determined by plate count and calculated as CFU per tissue. Experiments contained at least five mice per group, and data are representative of at least three experiments. **p < 0.01; ***p < 0.001.

To evaluate the TLT2 expression on macrophage during mycobacterial infection, we established a murine model by intravenous (i.v.) H37Rv or BCG infection. We analyzed the proportion of TLT2^+^F4/80^+^ cells in spleens at 21 days post infection. The percentage of TLT2^+^F4/80^+^ cells was increased after H37Rv or BCG infection (Figure 5A and Supplementary Figure 2A). The percentage of IL-6 expressing F4/80^+^ cells was significantly increased in TLT2^+^ F4/80^+^ cells compared with TLT2^–^F4/80^+^ cells (Figure 5B and Supplementary Figure 2B). Next, we evaluated whether TLT2 influenced CD4^+^ T cell-mediated anti-mycobacterial defense. We used recombinant mouse TLT2 (rmTLT2) as blocking agent. Mice were intraperitoneally injected (i.p.) with rmTLT2, followed by intravenous (i.v.) H37Rv or BCG infection 24 h later, injected with rmTLT2 again on day 7 post infection and all mice were sacrificed at 21 days post infection. Treatment with rmTLT2 decreased H37Rv- or BCG-induced IL-6 production and STAT3 phosphorylation in F4/80^+^ cells (Figures 5C–E and Supplementary Figures 2C–E). In rmTLT2-treated mice, increased IFN-γ production by CD4^+^ T cell were observed in the spleens and lungs compared with Nacl-treated mice (Figure 5F and Supplementary Figure 2F). Histopathological analysis revealed that rmTLT2 treatment alleviated the granuloma formation and attenuated inflammation of lung (Figure 5G and Supplementary Figure 2G). CFU assay showed that the bacterial load in the lungs and spleens of rmTLT2-treated mice were dramatically decreased (Figure 5H and Supplementary Figure 2H). These data indicated that rmTLT2 treatment induced Th1 response against H37Rv and BCG infection by inhibiting IL-6 production *in vivo*. Together, these results demonstrated that TLT2 enhances mice susceptibility to H37Rv and BCG infection.

## Discussion

PRRs play an important role in the regulation of innate or adaptive immune response against *Mtb* infection. However, the mechanism by which PRRs TLT2 is regulated during TB is not fully understood. Previous studies showed that macrophages up-regulate TLT2 expression in response to inflammation caused by injection of thioglycolate broth (16). Although TLT2 expression on B cell has high detectable level, it remained unchanged in response to inflammatory stimulation (16). Hashiguchi and colleagues have shown that TLT2 interacts with tumor-associated B7-H3 on T cell and promotes the proliferation of T cell (31). On the contrary, Leitner and colleagues showed that there is no evidence of B7-H3 and TLT2 interaction (32). Here, we demonstrated that TLT2 was up-regulated in human monocytes of ATB, while had no significantly difference on T cells or B cells. Therefore, we focused on the function of TLT2 expressed on myeloid cells.

Upon *Mtb* infection, macrophage can produce a series of cytokines and chemokines to regulate the immune response. Using siRNA to silence TLT2 expression, we found that TLT2 positively regulates *IL6* transcription and production on monocytes whereas it has no effect on other cytokines including *IL8*, *IL1B*, *IL10*, *TNF*, *TGFB*, and *GMCSF*. *Mtb*-induced IL-6 inhibits macrophage responses to IFN-γ (10) and IL-6 impairs intracellular killing of *Mtb* through altered lipid droplets homeostasis (33). IL-6 levels of ATB are associated with clinical symptoms and pathological changes (34). The presence of IL-6 during the differentiation of CD4^+^ T cells prevent the development of high IFN-γ-producing effector Th1 cells (5). In contrast, IL-6 induces Th2 differentiation in an IL-4 dependent manner (35). These mechanisms help IL-6 modulate the Th1/Th2 balance. The outcome of pathogenic *Mtb* infection can range from early asymptomatic clearance through latent infection to active disease. IFN-γ is critical to successful outcomes for chronic inflammatory disease such as TB due to its ability to enhance both anti-bacterial and pro-inflammatory response (36). Here, we showed that TLT2 suppresses Th1 response by promoting IL-6 production in monocyte, suggesting that TLT2 contributes to the progression of chronic TB infection. Blocking TLT2 *in vivo* with rmTLT2 can induce Th1 response, leading to higher bacterial killing. In this study, we revealed for the first time, the role of TLT2 expression in monocytes/macrophages during mycobacterial infection. Whether IL-6 regulation by TLT2 described here also occurs in other bacterial infection remains unknown.

Our study used BCG and H37Rv for *in vitro* and *in vivo* experiments. In our previous studies, BCG vaccination can be a mycobacterial analog for *Mtb* in innate and adaptive immunity (37–40). In our study, we found that TLT2 expression was increased in ATB and BCG/H37Rv infected monocyte/macrophage. All participants in our study are adults with TST test negative, which excluded the effect of BCG vaccine. The expression of TLT2 on macrophages was higher in TB patients than HC. *In vitro* experiment, *Mtb* infection induced higher TLT2 expression than BCG infection. Furthermore, BCG induced higher IL-6 production in PBMCs than ESAT-6/CFP10, while H37Rv induced higher IL-6 production than BCG. Our results indicated that IL-6 up-regulation in mycobacterial infection was not only due to ESAT-6/CFP10. TLT2 inhibited Th1 response against *Mtb* and BCG infection by promoting IL-6 production *in vitro* and *in vivo*.

TLT2 lacks either the conserved transmembrane lysine residue or ITAM/ITIM within its own cytoplasmic domain (26). Instead, the cytoplasmic tail of human TLT2 contains two tyrosine. One tyrosine (Y297) forms a YxxC motif, which has been shown to recruit and activate STAT3 in the G-CSFR signaling (27, 28). The other tyrosine (Y315) forms a potential endocytosis motif, YxxV (41). The YxxV motif has also been implicated in the recruitment and activation of STAT3 in the MEN2A-RET oncogene (29). STAT3 tyrosine phosphorylation is critical for IL-6 production in response to inflammation (42). To demonstrate TLT2 regulation of IL-6 production through STAT3 activation, we used the STAT3 inhibitor, Stattic, to examine if STAT3 inhibition can reverse the IL-6 enhancement caused by TLT2 overexpression. In addition, our results showed that the tyrosine residue Y297 and Y315 of TLT2 cytoplasmic tail region are involved in STAT3 activation. SOCS3, one of the members of the SOCS family, binds both JAK and the cytokine receptor, forming a ternary complex (43). SOCS3 is a major regulator of STAT3 activation and inhibits STAT3-mediated signaling by different cytokines, growth factors and hormones (43, 44). However, not all STAT3-activating cytokine receptors can be regulated by SOCS3. Whether SOCS3 controls the TLT2-mediated STAT3 signaling has not been determined.

Our experiment on mice showed that *Mtb* infection induced TLT2 expression on macrophages *in vivo*. To assess the involvement of TLT2 in host defense against *Mtb* infection, we tested whether blocking the effect of TLT2 impacted Th1 immune response *in vivo*. As one of the conserved TREM gene cluster molecules, function of recombinant TLT2 *in vivo* has yet to be identified. However, using recombinant protein for the TREMs signaling blockade had been reported by our and other groups (45–49). In our opinion, recombinant TLT2 works as decoy receptor to prevent the binding of its ligand to membrane-bound TLT2 which means a competitive inhibition of TLT2 signaling by recombinant TLT2. To investigate whether rmTLT2 could inhibit signaling, we examined the effect of rmTLT2 on the production of IL-6 and activation of STAT3. Our results showed that treatment with rmTLT2 decreased *Mtb*-induced IL-6 production and STAT3 phosphorylation, which was consistent with *in vitro* experiments using siTLT2. Our data indicated that recombinant TLT2 could be used as the blocking agent to inhibit TLT2 signaling. Therefore, TLT2 promoted *Mtb*-induced IL-6 production and STAT3 activation *in vivo*. Our results also showed that rmTLT2 treatment promoted Th1 response against *Mtb* infection, which resulted in decreased bacterial load in the organs. According to previous results, we concluded that TLT2 suppressed Th1 response against *Mtb* infection by promoting IL-6 production *in vivo*.

In the present study, we explored a novel mechanism by which TLT2 promotes IL-6 production in monocyte. Our results demonstrate that TLT2 interacted with JAK/STAT3 and enhanced STAT3 phosphorylation. Moreover, TLT2 suppressed IFN-γ production by CD4^+^ T cells and intracellular killing of mycobacteria by promoting IL-6 production in monocyte/macrophage. These findings may provide a potential therapeutic approach and target for TB.

## Data Availability Statement

The raw data supporting the conclusions of this article will be made available by the authors, without undue reservation.

## Ethics Statement

The study was approved by the Ethics Committee of the Fifth Hospital of Sun Yat-sen University (No. 2019-L097-1). The patients/participants provided their written informed consent to participate in this study. The animal study was reviewed and approved by Ethics Committee of ZSSOM on Laboratory Animal Care (No. 2014-010).

## Author Contributions

JL and CC designed and conducted the experiments. ZH, DH, and HZ were involved in funding acquisition. YW and XZ were involved in methodology design. JL, HY, YW, and CC analyzed the data. JL, CC, and XH prepared the manuscript. JL, CC, HX, YX, JZ, and XH reviewed and revised the draft. All authors have read and approved the final manuscript.

## Conflict of Interest

The authors declare that the research was conducted in the absence of any commercial or financial relationships that could be construed as a potential conflict of interest.
