# Supplementary material for: TLT2 Suppresses Th1 Response by Promoting IL-6 Production in Monocyte Through JAK/STAT3 Signal Pathway in Tuberculosis
Source: Front Immunol. 2020 Sep 11;11:2031. doi: 10.3389/fimmu.2020.02031 (PMC7516513; doi:10.3389/fimmu.2020.02031)
Supplement: Supplementary file 1 [file Table_1.docx]

## Supplementary Figures


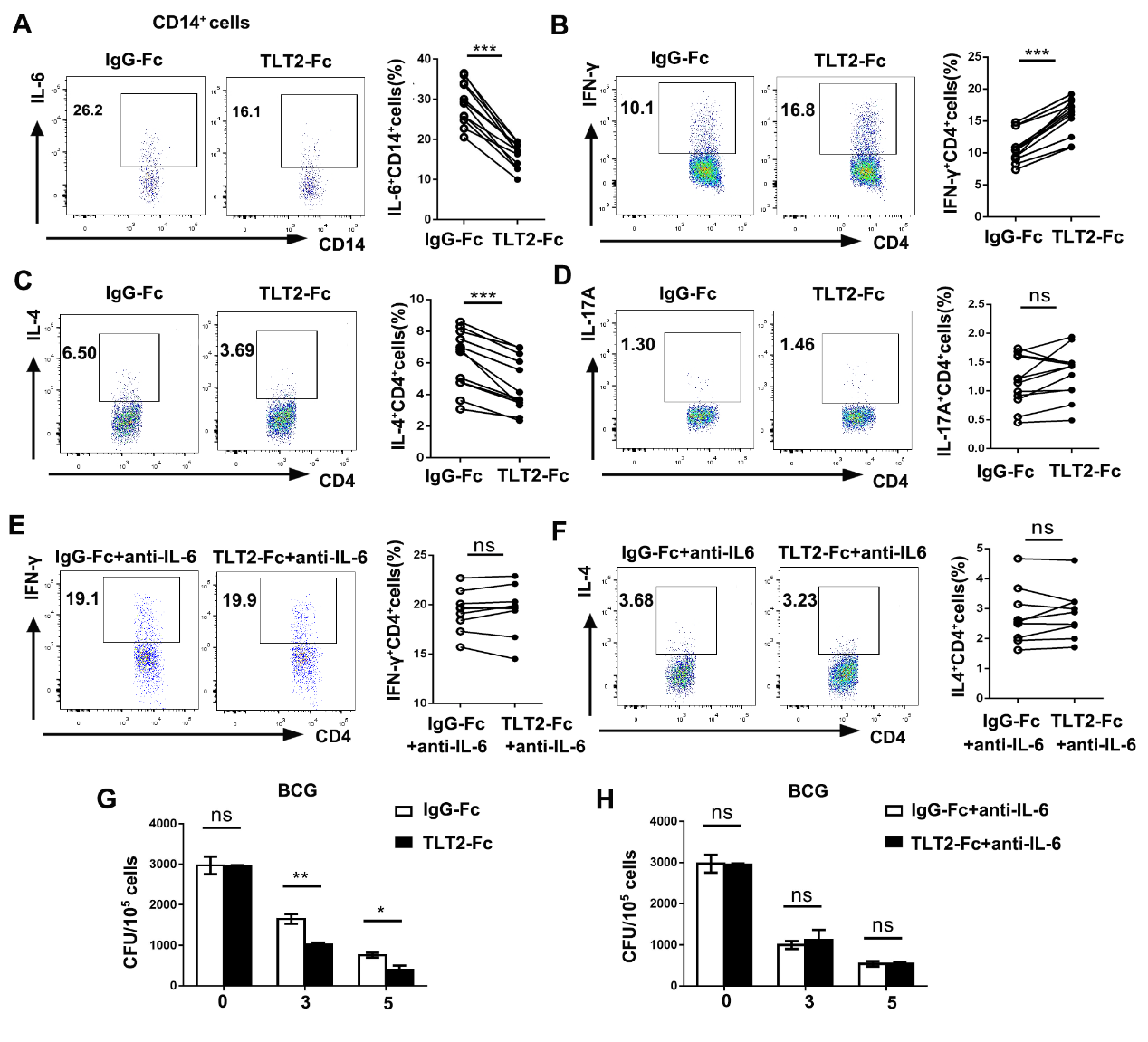


**Supplementary Figure 1. TLT2 expressing monocytes negatively regulated Th1 response against BCG infection.**

**(A)** CD14^+^monocytes isolated from PBMCs of the healthy controls were treated with recombinant chimeric human TLT2-Fc (4μg/mL) and human IgG1-Fc (4μg/ml) for 24hr followed by BCG infection at an MOI of 5 for 12hr. The TLT2^+^CD3^-^CD14^+^ cells were analyzed for the expression of IL-6 by flow cytometry. **(B-D)** Naïve CD4^+^T cell purified from human PBMCs using magnetic separation. CD4^+^T cell were activated with plate-bound anti-CD3 (1μg/ml) and anti-CD28 (1μg/ml) for 3 days co-culture with CD14^+^ monocytes in the presence of recombinant chimeric human TLT2-Fc (4μg/ml) and human IgG1-Fc (4μg/ml). CD14^+^ monocytes were pre-incubated with heat-killed BCG followed by co-cultured with CD4^+^ T cell. IFN-γ **(B)**, IL-4 **(C)** and IL-17A **(D)** expressions in CD4^+^ T cells were measured by flow cytometry, representative flow cytometric plots and the percentages of cells were shown. **(E, F)** Naïve CD4^+^ T cell co-cultured with CD14^+^ monocytes as described in B, but in the presence of neutralizing anti-IL-6 mAbs (10μg/ml) (anti-IL-6). IFN-γ and IL-4 expressions in CD4^+^ T cells were measured by flow cytometry, representative flow cytometric plots and the percentages of cells were shown. **(G)** Monocytes were infected with BCG at an MOI of 10 for 2hr. After washing, BCG-infected monocytes were cultured with T cells in the presence of anti-human IgG1-Fc or anti-human TLT2-Fc. CFU were calculated on Day 0, 3, 5. **(H)** BCG-infected monocytes were cultured with T cells as described in G, but in the presence of anti-IL-6 mAbs. CFU were calculated on Day 0, 3, 5. Data are a representative of at least three independent experiments. *, *p*<0.05; **, *p*<0.01; ***, *p*<0.001.


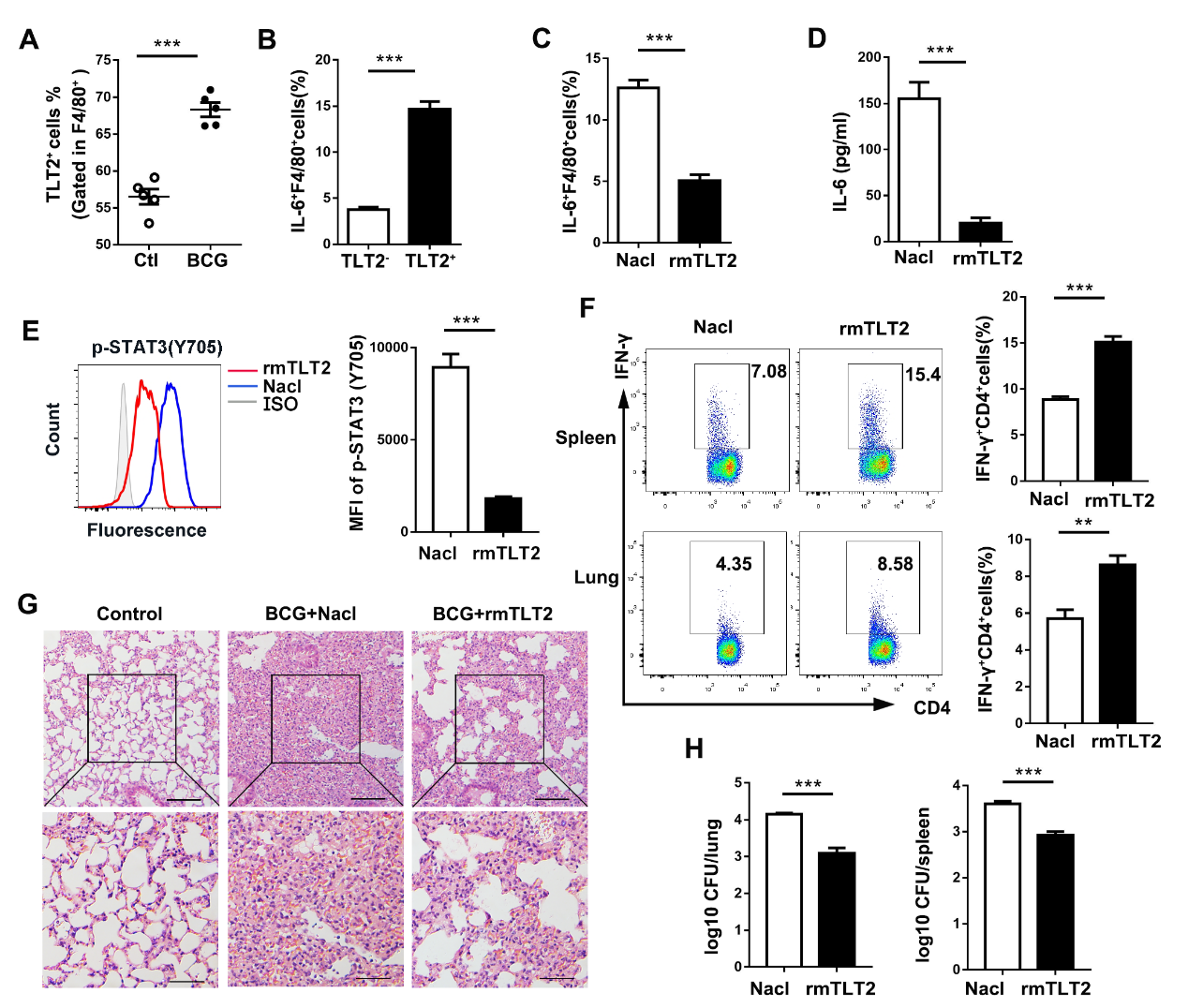


**Supplementary Figure 2. TLT2 inhibited Th1-mediated host defense against BCG infection *in vivo*.**

**(A)** C57BL/6 mice were intravenously (i.v.) injected with 1x10^6^ CFU of BCG. The proportion of TLT2^+^F4/80^+^ cells was assessed by flow cytometry in the spleens at 21 days post-infection. **(B)** IL-6 expression of spleen in TLT2^+^ or TLT2¯F4/80^+^ cells were analyzed by flow cytometry. Mice were intraperitoneally (i.p.) injected with recombinant mouse TLT2 (rmTLT2) for 24hr, followed by intravenous (i.v.) BCG infection. At 21 days post infection, the lungs and spleens were collected and analyzed for the following tests. **(C)** The frequency of splenic IL-6^+^ F4/80^+^ cells were determined by flow cytometry. **(D)** Protein level of IL-6 in serum was determined by ELISA. **(E)** STAT3 (Tyr705) phosphorylation in F4/80^+^ cells were analyzed by flow cytometry. **(F)** Percentages of IFN-γ producing splenic and pulmonary CD4^+^ T cells were analyzed by flow cytometry. **(G)** Lung sections were stained with hematoxylin-eosin (H&E) and checked for histopathology under microscope. **(H)** Bacteria burden in the lungs and spleens were determined by plate count and calculated as CFU per tissue. Experiments contained at least five mice per group, and data are representative of at least three experiments. **, *p* < 0. 01; ***, *p* < 0.001.
